# Radio-Frequency Localization of Multiple Partial Discharges Sources with Two Receivers

**DOI:** 10.3390/s18051410

**Published:** 2018-05-03

**Authors:** Guillermo Robles, José Manuel Fresno, Juan Manuel Martínez-Tarifa

**Affiliations:** Department of Electrical Engineering, Universidad Carlos III de Madrid, Avda, Universidad, 30, Leganés, 28911 Madrid, Spain; jfresno@ing.uc3m.es (J.M.F.); jmmtarif@ing.uc3m.es (J.M.M.-T.)

**Keywords:** localization, signal characterization, partial discharges, radio-frequency detection, condition monitoring, antennas, acoustic detection

## Abstract

Spatial localization of emitting sources is especially interesting in different fields of application. The focus of an earthquake, the determination of cracks in solid structures, or the position of bones inside a body are some examples of the use of multilateration techniques applied to acoustic and vibratory signals. Radar, GPS and wireless sensors networks location are based on radiofrequency emissions and the techniques are the same as in the case of acoustic emissions. This paper is focused on the determination of the position of sources of partial discharges in electrical insulation for maintenance based on the condition of the electrical equipment. The use of this phenomenon is a mere example of the capabilities of the proposed method but it is very representative because the emission can be electromagnetic in the VHF and UHF ranges or acoustic. This paper presents a method to locate more than one source in space with only two receivers, one of them in a fixed position and the other describing a circumference around the first one. The signals arriving from the different sources to the antennas are first separated using a classification technique based on their spectral components. Then, the individualized time differences of arrival (TDOA) from the sources collected at different angles describe a function, angle versus TDOA, that has all the geometric information needed to locate the source. The paper will show how to derive these functions for any source analytically with the position of the source as unknown parameters. Then, it will be demonstrated that it is possible to fit the curve with experimental measurements of the TDOA to obtain the parameters of the position of each source. Finally, the technique is extended to the localization of the emitter in three dimensions.

## 1. Introduction

The localization of emitting sources is an interesting research topic which covers several fields, from earthquake detection, [1], to RADAR and global positioning systems applied to the remote control of autonomous vehicles [2] or indoor positioning systems [3]. All these fields can use multilateration techniques applied to radio-frequency, acoustic and vibration signals which are acquired with several fixed sensors. Generally, when a pulse is radiated from a source, the wave will arrive to two receivers at different times. One of the advantages of measuring these time differences of arrival or TDOA is that a common clock is not required as in other localization techniques based on the time of arrival (TOA) of the pulse to the receiver. With two sensors, all the possible points in space that would give the same TDOA describe a hyperboloid [4]. Using two more independent receivers and calculating the intersection of the hyperboloids using all possible pairs of antennas would give the position of the source in three dimensions. Therefore, the spatial localization of emitters using multilateration techniques can be solved with at least four receivers.

In this work, the pulsed sources under study will come from a phenomenon known as partial discharges (PD) which are small-energy ionizations taking place within insulation materials revealing accelerated ageing [5]. Nevertheless, the results obtained here are not restricted to this type of pulsed source, since the same strategy could be applied to other types of signals whose source needed to be localized. It is well known that the detection of PD can be used to prevent premature failures of power cables and electric machines [6,7,8,9]. The interest in this technique is increasing since it can give indications on the insulation status with the equipment in service. In the particular case of open-air substations, the radio-frequency (RF) radiation emitted by PD can be used to detect and locate the imperfections, giving valuable information for condition monitoring [10,11,12].

There are other methods for localization based on the received signal strength (RSS) [13,14,15,16] or the angle of arrival (AOA) [17,18,19]. The use of the received signal strength indicator (RSSI) requires a previous mapping of the area to survey using a known source of discharges which sometimes would require putting the substation out of service. The sparker is placed in all points of a predefined grid and the power of the signal is recorded with a set of receivers. When a PD occurs in that mapped area, the signal acquired by the receivers is compared with the grid to set the approximate position of the source. The accuracy on the localization of the source depends on the density of data recorded in the grid and the previous elimination of all partial discharges that occurred during the characterization of the area. Concerning the calculation of the AOA, the usual approach is to use four receivers in the VHF and UHF bands or ultrasonic range forming and array of 2×2 elements. This array is virtually expanded to increase the resolution and then, the direction of the emission of the PD is computed with a multiple signal classification (MUSIC) algorithm [19].

Typically, the localization of PD sources using RF signals with multilateration techniques has been done through a system of four omnidirectional antennas [20]. The position of the receivers and TDOA of the pulses arriving to these sensors form a system of non-linear equations that can be solved to locate the emitter [21]. However, in the RF range the sampling frequencies ought to be high to have sufficient spatial resolution so the acquisition systems have a fairly high cost. It seems evident that a reduction in the number of receivers could help in the reduction of the cost of the instrumentation, mainly in sensors and high-speed digitizing channels. In this paper, the authors face this problem of moving at least one of the receivers with the aim of reducing the number of sensors using the known position of their relative movement to obtain the needed TDOA for the localization of the sources. Therefore, the authors propose a system for spatial localization of PD sources by means of two sensors mechanically linked by a rod. In order to get the necessary number of different TDOA for the localization, one of the sensors is rotated fixing the other one as a geometric reference. The mathematical function relating the relative distance between the two sensors, the rotation angle of the moveable one, and the TDOA will be derived in the paper showing good agreement with the experimental results. This approach was firstly applied in [22] obtaining good results for one source in the same plane as the antennas. In this paper, the method has been extended to two simultaneous PD sources and the localization of the sources in 3D. The casuistic when two or more sources are active is already complicated with four antennas, so reducing the number of antennas poses an additional challenge. This is tackled in the manuscript separating the origin of the signals based on their spectral signature using power ratio (PR) maps, a classification method based on the differences of power in certain bands of frequency [12,23].

The paper is structured in the following sections. First, the mathematical and geometrical principles of the method are explained in Section 2. Then, the problem of localizing more than one source is faced in Section 3 using the classification maps based on the spectral signature of the signals introduced in Section 4. Section 5 describes the measuring setup, the instrumentation and the experimental measurements. The algorithm and the method are extended to the localization of more than one source in three dimensions in Section 6. Finally the conclusions and insights of the work done in the paper are drawn in the last section.

## 2. Localization Basis

**Figure 1:**
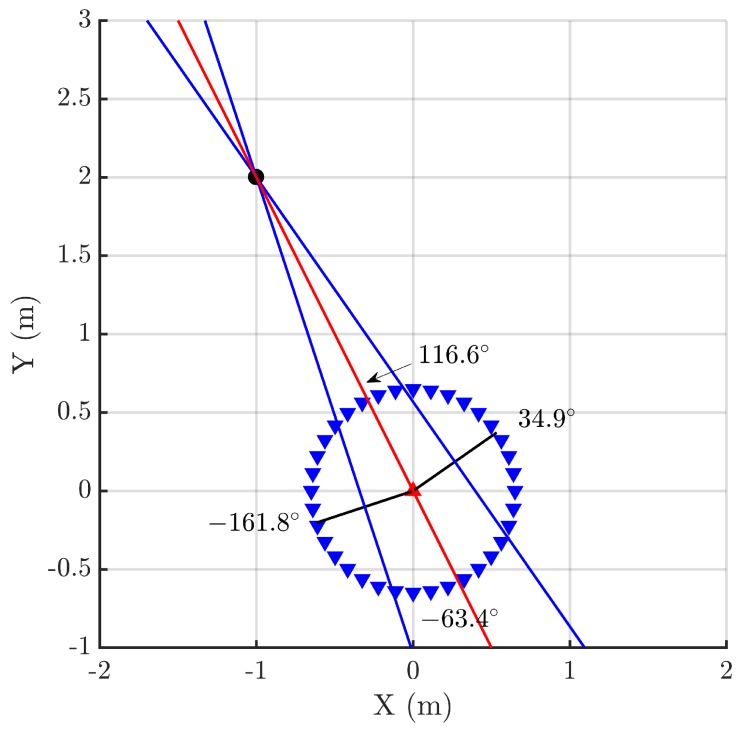
Different positions representative of antenna 2 marked with triangles (▼) when turned around the fixed antenna 1 (▲).

The proposed method is based on the information of the arrival of pulsed signals to two sensors, in this case, two simple omnidirectional antennas [24], as a function of their relative position with respect to the source. The TOA from the source to the antenna *i*, $t_{i}$, is not known since the position of the source has not yet been determined. Therefore, the localization relies on the time differences of arrival, $t_{12}=t_{1}-t_{2}$ of the emission to the antennas as in all multilateration techniques. The novelty of this work is presented henceforth. The distance between antennas is fixed, attaching them to the ends of a shaft or rod. Then, both antennas can be moved jointly when the rig is rotated around the vertical axis passing through the middle point between the antennas or the antenna 1 (▲) can be fixed at the origin of a Cartesian coordinate system while the antenna 2 (▼) rotates around the antenna 1 as shown in Figure 1 where the source (⬤) is placed at (-1,2) m. Though both possibilities are valid, the latter configuration is preferable to moving both antennas simultaneously since one of the receivers is always placed in the same place. This will be the method followed in the rest of the paper. Moreover, this stationary antenna will give the needed reference for the spectral characterization of the pulses explained in Section 4. Additionally, there are two possible methods to determine the position of the source. One of them is based on the intersection of two lines perpendicular to the rod when $t_{12}=0$ and the other is based on the determination of the dependence of the rotation angle with the TDOA and fitting the curve to the experimental data. These two procedures are explained in the next sections.

### 2.1. Intersection of Lines

There are four characteristic positions of the antennas depending on certain values of the TDOA: two zero crossings, and maximum and minimum values. The maximum corresponds to the case when the rod is aligned with the direction of the source, therefore, antenna 1, antenna 2 and source are in the same line. Figure 1 shows this particular case when the antenna 2 is placed at $116.6^{\circ }$, then $t_{1}>t_{2}$ and $t_{12}>0$. There is also a minimum at the opposite angle, $-63.4^{\circ }$, because there are two positions where the three elements are aligned. In this case, the relative locations of antenna 1 and antenna 2 are swapped and the TDOA is negative, $t_{2}>t_{1}$ and $t_{12}<0$. The position of the antennas also crosses zero twice and this happens when the rod is perpendicular to the direction of the source because, in that case, both times of arrival to the antennas are the same, $t_{1}=t_{2}$ and $t_{12}=0$, black radii in Figure 1. These cases are more helpful for this technique because plotting the perpendicular lines at the angular positions defined by the two zero crossings and calculating the intersection of the two lines will determine and solve the position of the source. Figure 1 shows these locations at $34.9^{\circ }$ and $-161.8^{\circ }$ and the lines perpendicular to the rod that intersect at the source.

In summary, the procedure to determine the position of the source is straightforward. First, the TDOA at different angles in one turn are calculated analyzing the onset of the pulses of the incoming signals to both antennas using any of the algorithms presented in [25] which, in this work, is based on the Hinkley criterion. Then, the angles where the TDOA are zero, $\zeta_{1}$ and $\zeta_{2}$, are calculated interpolating the closest angles above and below zero TDOA because the rotation is done in discrete angles. The next step traces the line perpendicular to the rod at those angles passing through its middle point. Finally, the position of the source is calculated with the intersection of those lines.

### 2.2. Analytical Expression of the Periodic Function

**Figure 2:**
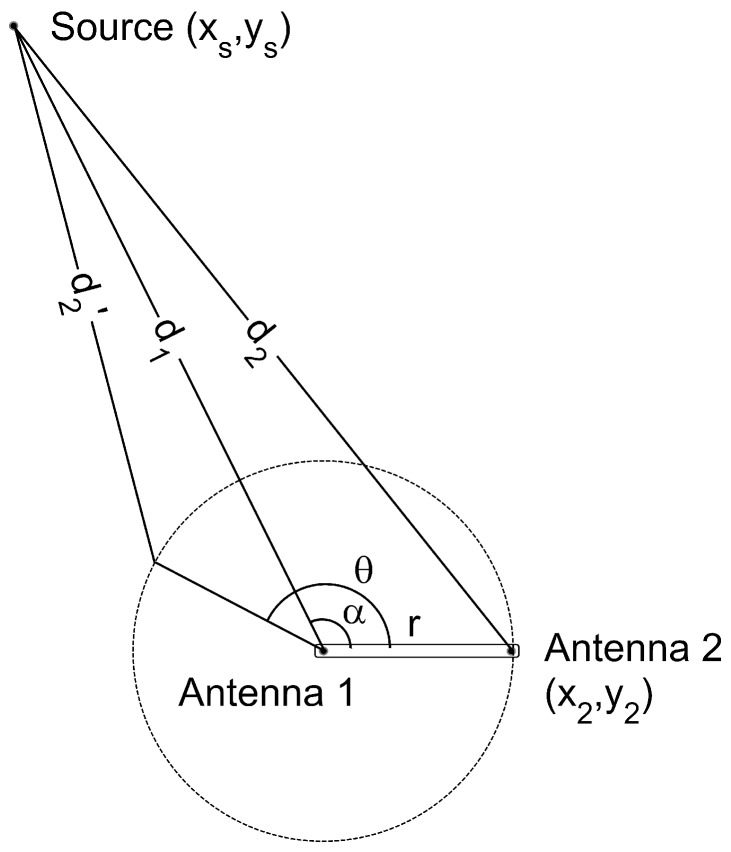
Source and antenna positions to determine the analytic expression of the periodic function θ vs. TDOA.

The analytical expression in two dimensions can be derived from the setup in Figure 2 where $d_{i}$ is the distance from the antenna *i* to the source. The antenna 1 is located at the origin of coordinates and the positions of the source and the antenna 2 are defined by their radius and azimuth in cylindrical coordinates $\left(d_{1},\alpha \right)$ and (r,θ), respectively. The TDOA in meters can be calculated subtracting the distances, $d=d_{1}-d_{2}$, which is better done in Cartesian coordinates:

(1) $$\begin{array}{ccc} \left(x_{s},y_{s}\right) & = & \left(d_{1}\cos\alpha ,d_{1}\sin\alpha \right)\\ \left(x_{2},y_{2}\right) & = & \left(r\cos\theta ,r\sin\theta \right)\\ {\vec{d}}_{2} & = & \left(d_{1}\cos\alpha -r\cos\theta ,d_{1}\sin\alpha -r\sin\theta \right) \end{array}$$

where $\left(x_{s},y_{s}\right)$ are the coordinates of the source, $\left(x_{2},y_{2}\right)$ the position of the antenna 2 and *r* the constant distance between antennas. Therefore, *d* is calculated as:

(2) $$d=d_{1}-\sqrt{{\left(d_{1}\cos\alpha -r\cos\theta \right)}^{2}+{\left(d_{1}\sin\alpha -r\sin\theta \right)}^{2}}$$

or, rearranging the terms, as:

(3) $$d=d_{1}-\sqrt{r^{2}+d_{1}^{2}-2rd_{1}\cos\left(\alpha -\theta \right)}$$

Equation (3) depends on the position of the source, determined by $d_{1}$ and α; the distance between antennas, *r* and the rotation angle, θ. The only unknown parameters are $d_{1}$ and α, so fitting the median of the experimental data for every angle to the analytical expression with a non-linear least squares algorithm will determine these parameters and, therefore, the position of the source.

**Figure 3:**
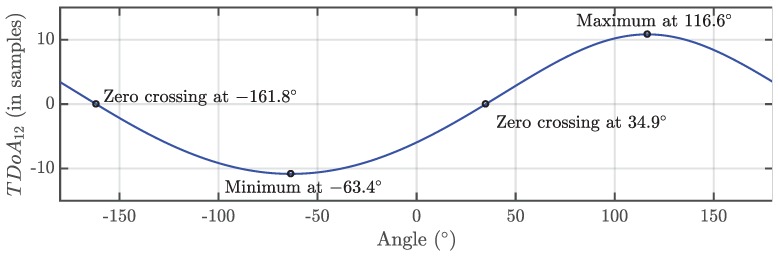
Periodic function representing the TDOA vs. the rotating angle. The zero-crossings correspond to the positions of the rig perpendicular to bearing of the source so the distances from the antennas to the source are the same. The maximum and minimum correspond to positions where the rig is parallel to this bearing so one of the antennas is at the farthest relative position with respect to the source and the other at the closest one.

Then, the process would consist of measuring the TDOA, *d*, at different angles completing a total turn to obtain the theoretical function plotted in Figure 3. The units of the TDOA are usually seconds but they can also be represented in meters multiplying time by the speed of light *c*, or samples, dividing time by the sampling time $T_{s}$. Concerning the choice of samples for the plot in the figure, one should bear in mind that the sampling frequency and the length of the rod are related and affect the sensitivity, as will be explained in Section 2.3.

There is an alternative formulation of Equation (3) represented in Cartesian coordinates that would give directly the coordinates of the source in this system:

(4) $$d=\sqrt{x_{s}^{2}-y_{s}^{2}}-\sqrt{{\left(x_{s}-x_{2}\right)}^{2}-{\left(y_{s}-y_{2}\right)}^{2}}$$

### 2.3. Choosing the Separation Distance

The separation between antennas is a critical parameter that will determine the accuracy of the calculated position of the source. The maximum time difference of arrival $d_{max}$, which is also the maximum and minimum of the analytic expression in Equation (3), can be derived considering that cos(α-θ)=1, or α=θ. In that case, the square root would be minimum and the maximum TDOA in meters would be:

(5) $$d_{max}=d_{1}-\sqrt{r^{2}+d_{1}^{2}-2rd_{1}}=d_{1}-\sqrt{{\left(d_{1}-r\right)}^{2}}=r$$

This result is expected since when the antennas are aligned (α=θ) and in the same plane as the source, the relative distance between the antennas and the source would be maximum and equal to the distance between them, *r*. This parameter is directly related to the sampling frequency and the sensitivity of the rig. In effect, since the sampling time determines the minimum measurable distance, the minimum step of *d* would be one sample or $cT_{s}$ meters. There is no point in selecting a small step in the rotation angle when the sampling frequency is low because the resolution in the vertical axis of Figure 3 would also be low. We can overcome this issue setting the length of the rod considering that we need to have a number of discrete steps of TDOA in the vertical axis in Figure 3 such as we can reach a sufficiently high resolution in the fitting of the curve. It has been considered that having 10 steps or samples up to the maxima of the curve is adequate, therefore, $d_{max}>10cT_{s}$ or $r>10cT_{s}$. This figure of merit together with the discrete steps in the rotation angle would ensure that the zero crossings can be determined with good accuracy.

## 3. Separation and Localization Strategy for Two Sources

Both possibilities of rotating the antennas commented on in Section 2 would give similar results for the position of the source, however, the difficulty arises when there is more than one source in different positions. Since the number of antennas has been reduced to two, there is only one time difference of arrival of the pulse to the receivers. If the measured signals come from two or more different sources, there is no easy way to know which TDOA would correspond to which sources. This problem has already been studied in [10] where the authors study the probability that a set of TDOA come from a certain source, however, we propose another approach based on the analysis of the shape of the pulse which simplifies the localization of multiple sources. It is well known that the shape of the signal received by the antenna depends on the radio-frequency pulse itself, the path from the source to the antenna, the radiometric characteristics of the antenna and interferences. In industrial environments, there can be metallic structures that distort the emitted pulse and the received signal would have a voltage increase due to the front wave and oscillations due to reflections and the antenna response. This poses additional problems in the determination of the pulse onset that have already been commented and solved in [21,26,27]. Since the fingerprint of the signal arriving to the antennas is highly dependent on the relative position of the source and the sensors and the environmental characteristics, the differences in the power spectral density of the signals can be used to determine their source of origin. If one of the antennas is stationary it would always receive signals with fingerprints associated to the sources resulting in a pattern database that will help in the association of signal and source. On the other hand, the mobile antenna would have the information of the time difference of arrival because it receives signals from the sources with a delay that is related to the path followed by the emission. Then, every acquisition has pairs of signals with information about the number of sources reported by antenna 1 and the TDOA of the emission of certain source reported by antenna 2. Since it is possible to classify the signals arriving to antenna 1 depending on their origin, the paired signals received by antenna 2 are automatically assigned to the same origin. The result is as many groups of pairs of signals as sources exist. Finally, once the signals are separated, every group is analysed individually to determine the position of the corresponding source applying any of the two methods presented in Section 2.

The alternative method introduced in Section 2 to acquire the signals moving both antennas would pose an important difficulty in determining the origin of the pulse since the information about the number of sources reported by the stationary antenna would be missing. In this case, there would be differences in terms of power spectral density between the signals received by the two antennas because the path followed from the source would be different. Then, the pair of signals should be grouped based on a statistical analysis of the TDOA finding similar time differences of arrival and hence determining the number of sources and their positions. As partial discharges are a stochastic phenomenon, an antenna can receive events in random times which could lead to mistakes in the association to the appropriate source. Unfortunately, this procedure is less reliable and produces worse results than if one of the antennas is stationary.

In summary, the process would start leaving antenna 1 fixed and rotating antenna 2 acquiring signals with both antennas every angle step. Then, classifying the signals in as many classes as emitting sources are using the information in the power spectral density with the power ratio maps classification method described in [23,28] and summarized in the next section. Finally, signals are extracted from the same source to determine their origin using multilateration techniques.

## 4. Power Ratio Maps Enhanced with Particle Swarm Optimization (PSO)

The power ratios map is applied to separate signals through their spectral power finding those bands of frequency where their spectra is different [28]. Let $\left[f_{1L},f_{2L}\right]$ and $\left[f_{1H},f_{2H}\right]$ be the limits for the low and high frequency intervals, respectively. The spectral power is calculated in those bands, referred to as the total power of the signal, so every signal would be parameterized with a power ratio at low frequencies, or PRL, and a power ratio at high frequencies, PRH:

(6) $$\begin{array}{ccc} PRL & = & \frac{\sum_{f=f_{1L}}^{f_{2L}}{\left|G(f)\right|}^{2}}{\sum_{f=0}^{f_{T}}{\left|G(f)\right|}^{2}} \end{array}$$

(7) $$\begin{array}{ccc} PRH & = & \frac{\sum_{f=f_{1H}}^{f_{2H}}{\left|G(f)\right|}^{2}}{\sum_{f=0}^{f_{T}}{\left|G(f)\right|}^{2}} \end{array}$$

where G(f) is the Fourier transform of the signal g(t) and $f_{T}$ is the highest frequency of interest of g(t).

Those signals corresponding to the same event would have similar spectra and so would be the parameters PRL and PRH. These signals would form a packed cluster when represented in a two dimensions scatter plot PRL versus PRH. Other events may present differences in these parameters, so the clusters would be plotted separately from the first one.

The selection of the frequency limits for the intervals can be done choosing the best set of frequencies that would group the same type of signals in *k* packed clusters and gives the largest separation between clusters. The clusters are selected using the k-means algorithm because it is manageable and easy to implement. Then, the distance between the centroids of clusters *i* and *j* and the spread of the clusters are simultaneously evaluated using the Mahalanobis distance $m_{ij}$. The frequency intervals are selected maximizing the minimum distance between the centroids so the objective function *M* would be:

(8) $$M=\max\min_{i\neq j}^{k}m_{ij}$$

In the case of two sources, k=2, and the Equation (8) can be simplified to $M=\max m_{12}$.

Particle swarm optimization (PSO) is then applied to obtain *M* through the selection of a valid set of intervals. PSO is a metaheuristic algorithm with hive intelligence based on the behaviour of animals such as birds or fish in search for food. The algorithm starts deploying a set of particles in the solutions space and moving them towards an optimum with information of the achievements of the swarm. The dimension of the particles is related to the five parameters used to define the PR map, $\left[f_{1L},f_{2L}\right]$, $\left[f_{1H},f_{2H}\right]$ and $f_{T}$. In every iteration *l* the algorithm stores the position of every particle for its personal best solution for *M* so far, $P_{n,b}$, and the position of the particle that has achieved the overall best solution for *M*, $P_{b}$. The movement of particle *n* towards the solution for *M*, $v_{n}$, is calculated with the following standard PSO algorithm:

(9) $$\begin{array}{ccc} v_{n}\left(l+1\right) & = & v_{n}\left(l\right)+c_{1}U_{1}\left(0,1\right)⊗\left[P_{n,b}\left(l\right)-P_{n}\left(l\right)\right]\\ & + & c_{2}U_{2}\left(0,1\right)⊗\left[P_{b}\left(l\right)-P_{n}\left(l\right)\right],\\ P_{n}\left(l+1\right) & = & P_{n}\left(l\right)+v_{n}\left(l+1\right), \end{array}$$

where $U_{1}\left(0,1\right),U_{2}\left(0,1\right)\in {\left[0,1\right]}^{5}$ are five-dimensional random vectors, with each component independently drawn from a uniform distribution between 0 and 1. Both $U_{1}\left(0,1\right),U_{2}\left(0,1\right)$ randomize the movement of the particles towards their own best and the swarm’s best, respectively. The operator ⊗ multiplies the random numbers by the five coordinates component by component. The parameters $c_{1}$ and $c_{2}$ describe the balance between the personal influence of the particle and the social influence in the search of the solution. After a certain number of iterations the algorithm converges towards a set of frequencies that maximizes the distance between the two clusters. The outcome of the application of these algorithms is *k* sets of signals coming from *k* different sources that can be studied individually to determine the time differences of arrival to the two antennas.

## 5. Setup and Analysis of the Measurements

**Figure 4:**
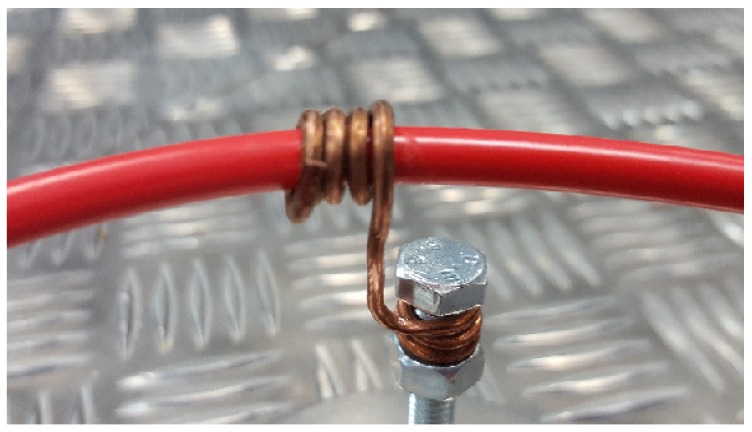
Detail of the section of the high voltage cable (in red) with the wound wire to ground.

**Figure 5:**
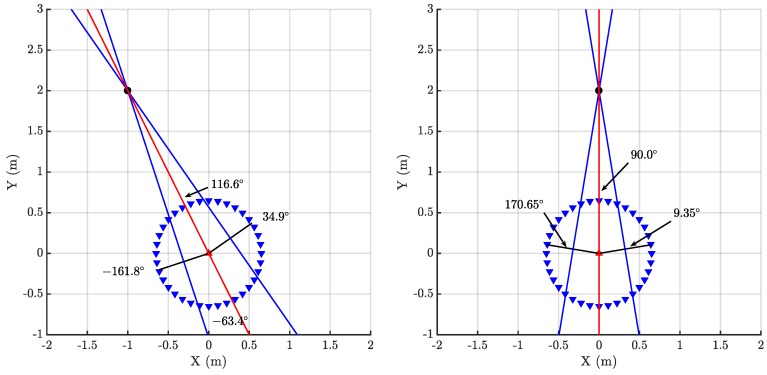
Positions of source 1 (**left**) and source 2 (**right**) and the theoretical zero-crossing and bearing angles.

Partial discharges were activated winding a grounded enameled copper wire around an unshielded high-voltage cable, see Figure 4. When the voltage is applied there would be a high electric field divergence in that area that would create surface discharges. This procedure is repeated in another section of the cable so there would be two similar sources of partial discharges. The antennas are two simple omnidirectional monopoles 10 cm in length that have good response in the frequency range where partial discharge emits energy which for these test objects is below 400 MHz [24]. In the measurements in two dimensions, the stationary antenna is placed at the origin of a Cartesian coordinate system. The positions of the two emitters are $S_{1}=\left(-1,2\right)$ m and $S_{2}=\left(0,2\right)$ m in Cartesian coordinates and $S_{1}=2.24∠116.6^{\circ }$ m and $S_{2}=2∠90^{\circ }$ m in polar coordinates, Figure 5. The antennas are in the same plane attached to a rod r=0.65 m long and connected to a high-speed digitizing oscilloscope through coaxial cables 5 m long. The sampling frequency was set to 5 GS/s so the sampling time is $T_{s}=200$ picoseconds. The distance between the antennas fulfills the relationship $r>10cT_{s}$ since $10cT_{s}=0.6$ m so there will be at least 10 TDOA in samples between the signals arriving to the antennas when the antennas and the source are in the same bearing. The antennas were rotated 360 degrees every 10 degrees. To have a statistically reliable TDOA, the oscilloscope acquired 500 signals for every angle step.

**Figure 6:**
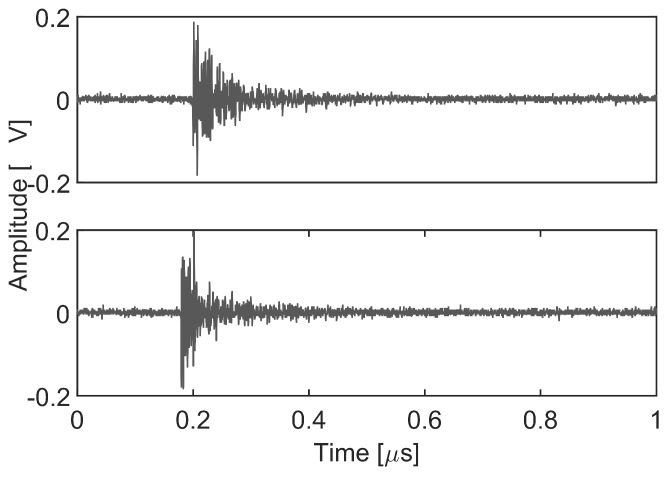
Two characteristic PD signals from the two sources acquired by the antenna 1.

**Figure 7:**
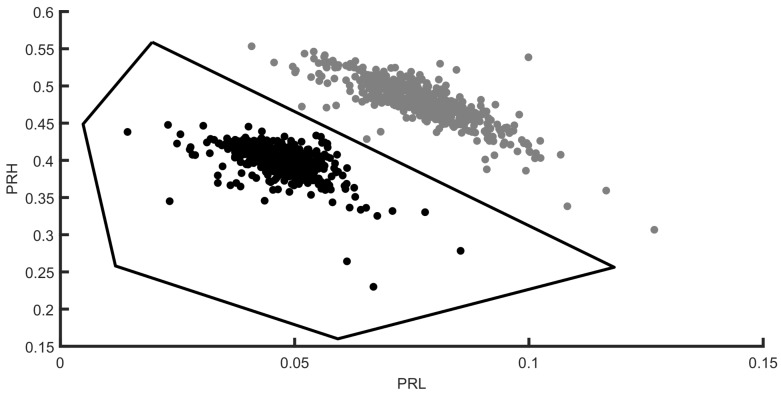
Signals received by antenna 1 represented in a PR map where the differences in the power spectral density corresponding to two sources distributes the signals into clusters.

**Figure 8:**
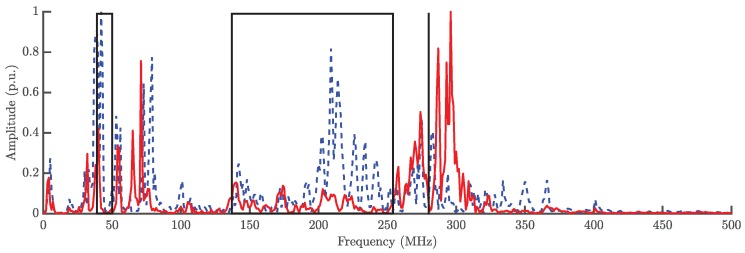
Average power spectral density of the signals captured by the antenna 1 and the selected intervals that maximize the distance between clusters in the PR map. The solid line corresponds to dark cluster and the dashed plot to the grey cluster.

Figure 6 presents two PD pulses, one for each source, captured with the antenna 1. Once the signals have been acquired, the analysis process starts calculating their spectral power density arriving to the stationary antenna. Bear in mind that this antenna would receive signals in which the signature of the environmental characteristics of the installation is stable and distinctive for every source while the moveable antenna will be used only to find the TDOA. Figure 7 shows a PR map with two clusters corresponding to the signals arriving to this antenna from two different sources. The average power spectral density of the signals from each cluster is represented in Figure 8 together with the selected intervals in the low and the high frequency ranges to separate the sources. These bands, PRL and PRH, are selected automatically using PSO to maximize the separation of the clusters as explained in Section 4 and results $f_{1L}=39$ MHz, $f_{2L}=50$ MHz, $f_{1H}=137$ MHz, $f_{2H}=254$ MHz and $f_{T}=280$ MHz. Both intervals select more power from the dashed line than from the solid one. This places the dots in the PR map with higher PRH and PRL values obtaining the grey cluster. Additionally, the black cluster has only around 5% of the total power in the low frequency interval, PRL, which places it at the left side of the other cluster. Notice that the differences in the spectra are due to the fact that the paths that follow the signals are not the same since the physical effect of the discharges is quite similar.

The polygon in Figure 7 represents the boundaries of the first cluster selected with Matlab for the analysis of the position of one of the sources. As explained in Section 3 the signals received by antenna 1 are paired with the signals of antenna 2 thanks to the two channels in the acquisition system; so, selecting one of the clusters corresponding to one of the sources will give the signals from that source received by antenna 2.

**Figure 9:**
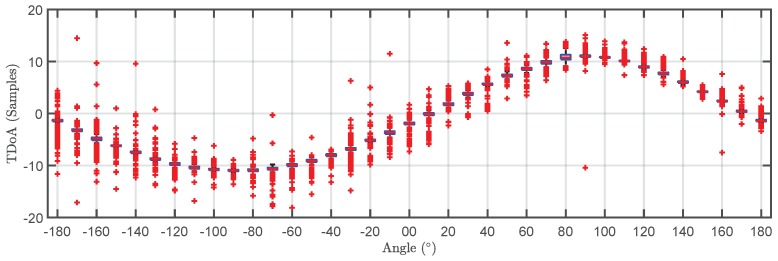
Box plot of the time differences of arrival versus angle of rotation for the pulses generated by source 1 (black cluster).

**Figure 10:**
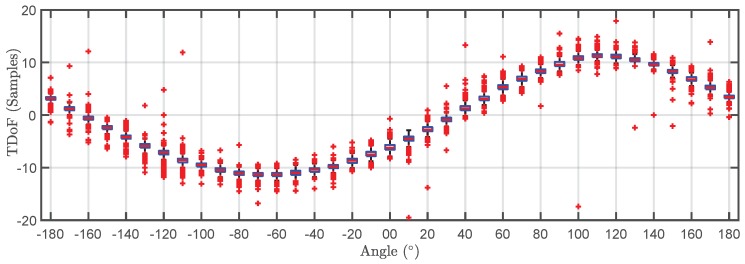
Box plot of the time differences of arrival versus angle of rotation for the pulses generated by source 2 (grey cluster).

**Figure 11:**
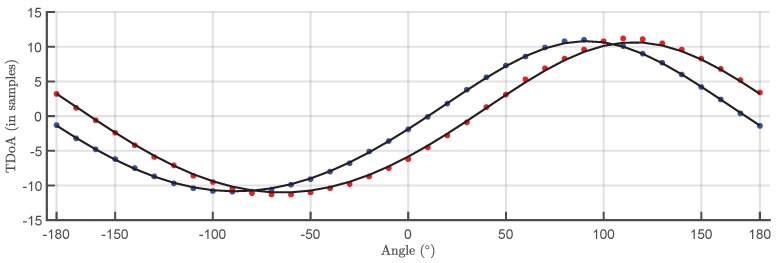
Median of all TDOA versus rotation angle and fitting function. Blue plot corresponds to source 1 and red plot to source 2.

The TDOA for the 500 pulses in each cluster are plotted versus the angle of rotation in a box plot in Figure 9 and Figure 10. The bottom and top of the boxes in blue represent the boundaries for 25% to 75% of all the values obtained for the TDOA while the red line inside the box represents the median value. The red crosses outside the box are estimations of the TDOA given by the algorithm for some of the pulses that do not have clear onsets. These cases give TDOA outside the boundaries of the box so they can be considered statistical outliers though they are also considered in the calculation of the median value and the boundaries of the box. Admitting that it may be thought that the number of outliers is high, it can be clearly seen that the boxes are very narrow which means that most of the results are concentrated around the median. Figure 11 shows the fitting of the periodic functions considering only the median of all TDOA in every angle of rotation. The numerical results for the source 1 after the fitting with Equation (4) are ${\hat{x}}_{s1}=-0.04$ m and ${\hat{y}}_{s1}=2.11$ m while the exact values, considering the layout in Figure 5 were $x_{s1}=0$ m and $y_{s1}=2$ m. The shift in Euclidean distance between the actual position of the source and the estimation is 12 cm. The fitting in Figure 11 is assessed with the mean square error considering the median of the TDOA yielding $\epsilon_{1}=0.1061$ samples or $\epsilon_{1}=0.64$ in centimeters which are also very low. The results for the source 2 are ${\hat{x}}_{s2}=-0.99$ m and ${\hat{y}}_{s2}=2.07$ m while the actual values are $x_{s1}=-1$ m and $y_{s1}=2$ m which are also very close to the estimated position. The error between the actual source position and the estimated one is 7.1 cm while the mean square error of the fitting considering the median of the TDOA is $\epsilon_{2}=0.3627$ samples or $\epsilon_{2}=2.2$ cm. As additional information, the estimated zero crossings obtained with the method presented in Section 2.1 for source 1 and 2, respectively, are $\zeta_{11}=34.17^{\circ }$, $\zeta_{12}=-161.18^{\circ }$ and $\zeta_{21}=8.18^{\circ }$, $\zeta_{22}=167.5^{\circ }$ which are very close to the theoretical ones in Figure 5. Though the error is well below $1^{\circ }$ in both angles in the case of source 1, the intersection of the lines is shifted from its actual position: ${\hat{x}}_{s1}=-1.09$ m and ${\hat{y}}_{s1}=2.18$ m which corresponds to an error of 20.1 cm. In the case of the source 2, ${\hat{x}}_{s2}=0.08$ m and ${\hat{y}}_{s2}=1.81$ m placing the source at position shifted 20.6 cm from the actual one.

## 6. Localizing in Three Dimensions

**Figure 12:**
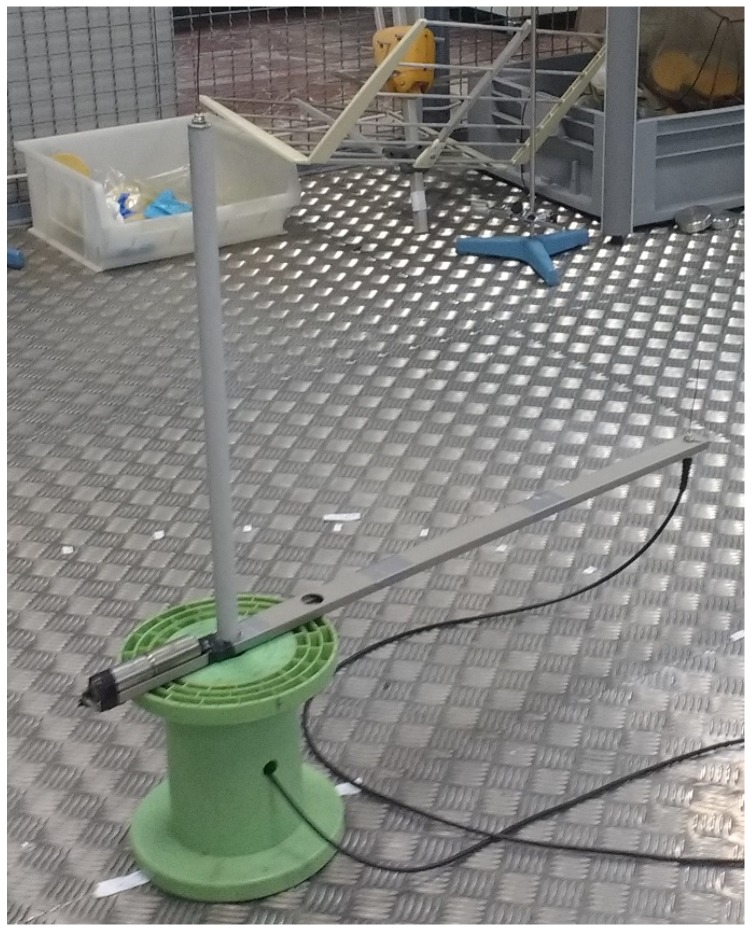
Antenna layout for three dimensional localization.

The extension of the method to locate sources in three dimensions is straightforward, placing one of the antennas at a different height so there can be a shift of the TDOA received by that antenna with the information in the *Z* axis. The easiest setup would result in elevating the stationary antenna as shown in Figure 12. The method in Section 2.1 will be discarded henceforth since the results obtained in 2D for two sources have not been very accurate. Moreover, if one of the antennas is in a different plane, the zero crossings in Figure 3 would not correspond to a position in which the rod is perpendicular to the direction of the source.

**Figure 13:**
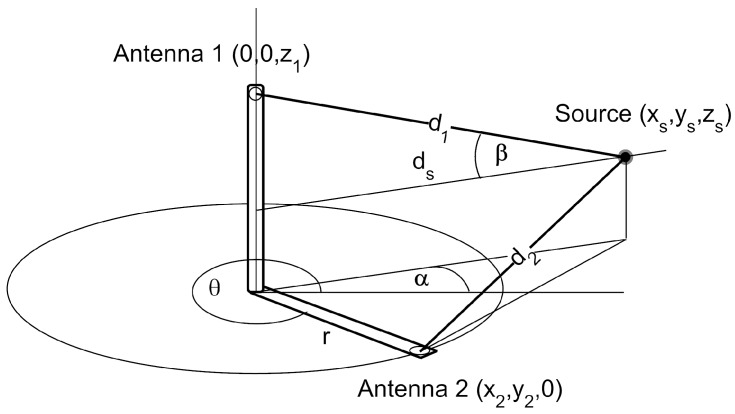
Geometric representation of the setup with the main parameters of the equations.

The equations derived in Section 2.2 have to be modified to use three components. The starting point also considers that the TDOA in meters can be calculated subtracting the distances, $d=d_{1}-d_{2}$, where $d_{1}$ and $d_{2}$ are again the distances from antennas 1 and 2 to the source, respectively. From Figure 13, $d_{s}$ can be defined as the distance of the source to the vertical axis *Z*, $d_{s}=d_{1}\cos\beta$, with $\beta =\arcsin[\left(z_{1}-z_{s}\right)/d_{1}]$. Then, the equations can be written as:

(10) $$\begin{array}{ccc} \left(x_{s},y_{s},z_{s}\right) & = & \left(d_{s}\cos\alpha ,d_{s}\sin\alpha ,z_{s}\right)\\ \left(x_{1},y_{1},z_{1}\right) & = & \left(0,0,z_{1}\right)\\ {\vec{d}}_{1} & = & \left(d_{s}\cos\alpha ,d_{s}\sin\alpha ,z_{s}-z_{1}\right)\\ \left(x_{2},y_{2},z_{2}\right) & = & \left(r\cos\theta ,r\sin\theta ,0\right)\\ {\vec{d}}_{2} & = & \left(d_{s}\cos\alpha -r\cos\theta ,d_{s}\sin\alpha -r\sin\theta ,z_{s}\right) \end{array}$$

where $\left(x_{s},y_{s},z_{s}\right)$ are the coordinates of the source, $\left(x_{1},y_{1},z_{1}\right)$ and $\left(x_{2},y_{2},z_{2}\right)$ the positions of antennas 1 and 2 and *r* is the length of the rod that connects antenna 2 to the base of antenna 1. Therefore, *d* is calculated as:

(11) $$d=d_{1}-\sqrt{{\left(d_{s}\cos\alpha -r\cos\theta \right)}^{2}+{\left(d_{s}\sin\alpha -r\sin\theta \right)}^{2}+z_{s}^{2}}$$

or,

(12) $$d=d_{1}-\sqrt{d_{s}^{2}+r^{2}-2rd_{s}\cos\left(\alpha -\theta \right)+z_{s}^{2}}$$

In these equations, there are only three unknown parameters: $d_{1}$, α and $z_{s}$ since $d_{s}$ is related to $d_{1}$ and θ is the rotation angle.

**Figure 14:**
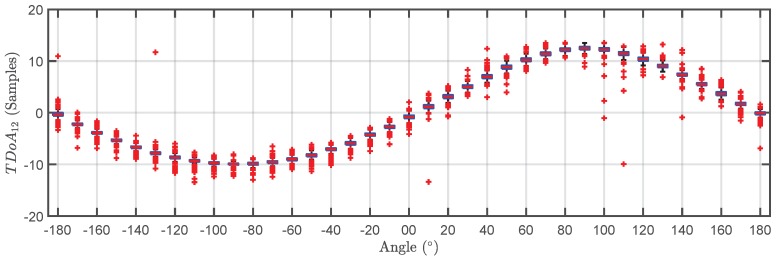
Box plot of the time differences of arrival versus angle of rotation for the pulses generated by source 1 in 3D localization.

**Figure 15:**
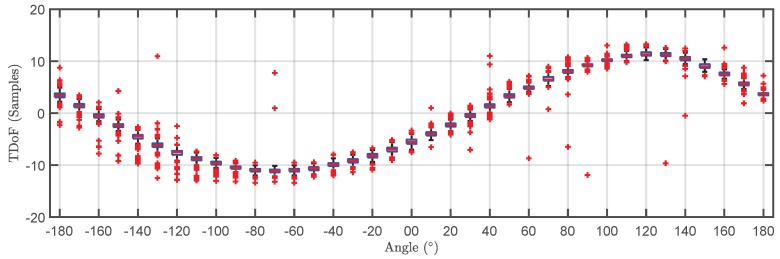
Box plot of the time differences of arrival versus angle of rotation for the pulses generated by source 2 in 3D localization.

**Figure 16:**
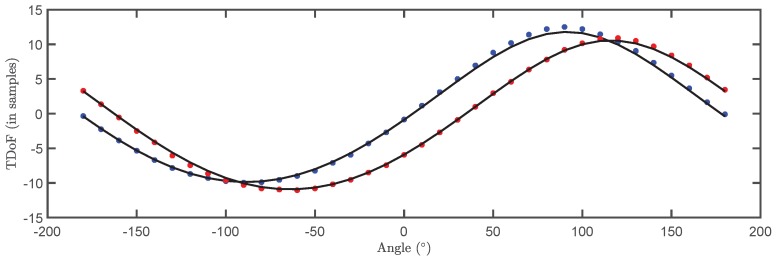
Median of all TDOA versus rotation angle in 3D localization with the fitting function. Blue plot corresponds to the fitting function for source 1 and red plot to source 2.

The next steps are the same as in the case of the localization in 2D: The signals from two sources received at the stationary antenna are separated using the PR classification map. This sets the correspondence between the events captured by the stationary antenna and the sources. Since every signal in the PR map is paired to another signal acquired by the movable antenna, it is possible to determine the TDOA for each source. These time differences are again estimated with the Hinkley criterion at different positions of antenna 2 and plotted versus the angle of rotation in a box plot. The results are presented in Figure 14 and Figure 15. Then, Equation (12) is used to fit the plot of median values of the TDOA for a complete rotation of the rig to obtain the plots shown in Figure 16. The resulting fitting parameters yield the Cartesian coordinates of the source using Equation (10).

There is also an alternative formulation of Equation (12) using directly Cartesian coordinates:

(13) $$d=\sqrt{x_{s}^{2}+y_{s}^{2}+{\left(z_{s}-z_{1}\right)}^{2}}-\sqrt{{\left(x_{s}-x_{2}\right)}^{2}+{\left(y_{s}-y_{2}\right)}^{2}+{\left(z_{s}-z_{2}\right)}^{2}}$$

In this case, the position of the source $\left(x_{s},y_{s},z_{s}\right)$ is readily obtained from the fitting. The results for the median TDOA have been fitted again with Matlab with a non-linear least squares algorithm using the relations in Equations (10) and (13).

The localization is satisfactory as shows Table 1 and the mean square error of the fitting is very low $\epsilon_{1}=0.2277$ samples or $\epsilon_{1}=1.37$ cm for the source 1 and $\epsilon_{2}=0.1247$ samples or $\epsilon_{2}=0.75$ cm for the source 2.

## 7. Conclusions

This paper proposes a new method to determine the position of pulsed sources such as the emission of partial discharges. The localization is based on the fact that the signals that arrive to a stationary antenna from different sources have different signatures. The fingerprint has information about the direct path from the source to the antenna, reflections and attenuation. This information is used to separate the sources and study the time difference of arrival to the receivers to determine their position. The other antenna is rigged to the stationary one and describes a circumference to have different time differences of arrival. The function that relates the TDOA with the rotation angle can be derived mathematically based on the geometry of the setup and the position of the sources. Then, the experimental data is fitted to this function to obtain the position of the sources. The usability of the method has been demonstrated for two sources in two and three dimensions obtaining good results. The TDOA are determined with the Hinkley criterion based on the change of the accumulated energy in the signals and this step is critical to have accurate results. The value of the selected TDOA in every rotation angle is based on the median value because there is a certain number of outliers that would render the average value useless. These outliers correspond to signals where the Hinkley criterion fails to find a correct value because the first frontwave of the pulse is not clear or the noise triggers an early picking of the onset of the pulse. In any case, the number of TDOA close to the correct value is high and the experimental functions are fitted accurately. A previous denoising of the signals with the discrete wavelet transform or the PR maps (such as in [12]) may help in improving the accuracy in the determination of the position of the source since the pickings of the onsets with the Hinkley criterion would be better. Another source of random error is the accurate positioning of the movable antenna because the movement is not still automatized. We expect to have this resolved with a stepper motor capable of rotating accurately less than one degree per step.

The practical application of this setup with two antennas in an open-air substation is straightforward since the area that occupies the rotating antenna is not large and would not interfere with the assets. The rig can be placed anywhere in the installation as long as the distances to the farther equipment stay inside the range of the antennas so the signal-to-noise ratio is appropriate to detect the discharges. It might be necessary to measure in several places if the signals acquired are highly distorted due to metallic structures in the line-of-sight. In each place, a complete rotation of the mobile antenna is recommended to have a good fitting with the theoretical curve. If the separation between sources is very short, the proposed setup would also be able to detect them as long as it is larger than the minimum TDOA which in the case of this paper is 6 cm. The problem when two sources are very close would be having two different propagation paths to distinguish the signals with the PR method. Therefore, two very close PD in the same side of an insulator, for instance, might follow a similar propagation path and the separation method may fail; although, two close PD on different sides of the insulator would be clearly identified.

## Figures and Tables

**Table 1:** Results of the estimation of parameters in the 3D localization. The theoretical position of the sources are: (0,2,0) m and (-1,2,0.3) m, for source 1 and 2, respectively.

	Source 1	Source 2
	$x_{s}=-0.050$ m	$x_{s}=-0.989$ m
	$y_{s}=2.073$ m	$y_{s}=2.027$ m
	$z_{s}=0$ m	$z_{s}=0.279$ m
Euclidean error	0.089 m	0.036 m

## References

[1] Lee K., Kwon H., You K. (2017). Laser-Interferometric Broadband Seismometer for Epicenter Location Estimation. Sensors.

[2] Moreau J., Ambellouis S., Ruichek Y. (2017). Fisheye-Based Method for GPS Localization Improvement in Unknown Semi-Obstructed Areas. Sensors.

[3] Xiong R., van Waasen S., Rheinlnder C., Wehn N. (2017). Development of a Novel Indoor Positioning System With mm-Range Precision Based on RF Sensors Network. IEEE Sens. Lett..

[4] Zou Y., Liu H., Xie W., Wan Q. (2017). Semidefinite Programming Methods for Alleviating Sensor Position Error in TDOA Localization. IEEE Access.

[5] Kreuger F. (1989). Partial Discharge Detection in High-Voltage Equipment.

[6] (2012). IEEE Guide for Field Testing and Evaluation of the Insulation of Shielded Power Cable Systems Rated 5 kV and Above.

[7] (2012). Rotating Electrical Machines—Part 27-2: On-Line Partial Discharge Measurements on the Stator Winding Insulation of Rotating Electrical Machines.

[8] Stone G.C., Sedding H.G., Chan C. (2018). Experience With Online Partial-Discharge Measurement in High-Voltage Inverter-Fed Motors. IEEE Trans. Ind. Appl..

[9] AJ C., Salam M., Rahman Q., Wen F., Ang S., Voon W. (2018). Causes of transformer failures and diagnostic methods—A review. Renew. Sustain. Energy Rev..

[10] Li P., Zhou W., Yang S., Liu Y., Tian Y., Wang Y. (2017). A Novel Method for Separating and Locating Multiple Partial Discharge Sources in a Substation. Sensors.

[11] Mishra D.K., Sarkar B., Koley C., Roy N.K. (2017). An unsupervised Gaussian mixer model for detection and localization of partial discharge sources using RF sensors. IEEE Trans. Dielectr. Electr. Insul..

[12] Robles G., Fresno J.M., Martínez-Tarifa J.M. (2015). Separation of Radio-Frequency Sources and Localization of Partial Discharges in Noisy Environments. Sensors.

[13] Xu Y., Zhou J., Zhang P. (2014). RSS-Based Source Localization When Path-Loss Model Parameters are Unknown. IEEE Commun. Lett..

[14] Zhang Y., Upton D., Jaber A., Ahmed H., Khan U., Saeed B., Mather P., Lazaridis P., Atkinson R., Vieira M.F.Q., Glover I.A. Multiple source localization for partial discharge monitoring in electrical substation. Proceedings of the 2015 Loughborough Antennas Propagation Conference (LAPC).

[15] Li Z., Luo L., Zhou N., Sheng G., Jiang X. (2017). A Novel Partial Discharge Localization Method in Substation Based on a Wireless UHF Sensor Array. Sensors.

[16] Li Z., Luo L., Sheng G., Liu Y., Jiang X. (2018). UHF partial discharge localisation method in substation based on dimension-reduced RSSI fingerprint. IET Gener. Transm. Distrib..

[17] Gao S., Zhang Y., Xie Q., Kan Y., Li S., Liu D., Lü F. (2017). Research on Partial Discharge Source Localization Based on an Ultrasonic Array and a Step-by-Step Over-Complete Dictionary. Energies.

[18] Wielandt S., Strycker L.D. (2017). Indoor Multipath Assisted Angle of Arrival Localization. Sensors.

[19] Yongfen L., Xiaohu X., Fei D., Xiao T., Yanming L. (2015). Comparison of DOA Algorithms Applied to Ultrasonic Arrays for PD Location in Oil. IEEE Sens. J..

[20] Zhu M.X., Wang Y.B., Liu Q., Zhang J.N., Deng J.B., Zhang G.J., Shao X.J., He W.L. (2017). Localization of multiple partial discharge sources in air-insulated substation using probability-based algorithm. IEEE Trans. Dielectr. Electr. Insul..

[21] Robles G., Fresno J.M., Sánchez-Fernández M., Martínez-Tarifa J.M. (2016). Antenna Deployment for the Localization of Partial Discharges in Open-Air Substations. Sensors.

[22] Fresno J.M., Robles G., Martínez-Tarifa J.M. (2018). Planar Localization of Radio-Frequency or Acoustic Sources with Two Receivers. Proceedings.

[23] Fresno J.M., Ardila-Rey J.A., Martínez-Tarifa J.M., Robles G. (2017). Partial discharges and noise separation using spectral power ratios and genetic algorithms. IEEE Trans. Dielectr. Electr. Insul..

[24] Robles G., Sánchez-Fernández M., Albarracín Sánchez R., Rojas-Moreno M., Rajo-Iglesias E., Martínez-Tarifa J. (2013). Antenna Parametrization for the Detection of Partial Discharges. IEEE Trans. Instrum. Meas..

[25] Robles G., Fresno J.M., Giannetti R. (2017). Ultrasonic bone localization algorithm based on time-series cumulative kurtosis. ISA Trans..

[26] Boker E., Levy A., Goldreich R. (2017). Mitigation of Multipath Distortions for TDOA-Based Geolocation. U.S. Patent.

[27] Bai F., Gagar D., Foote P., Zhao Y. (2017). Comparison of alternatives to amplitude thresholding for onset detection of acoustic emission signals. Mech. Syst. Signal Process..

[28] Robles G., Fresno J.M., Martínez-Tarifa J.M., Ardila-Rey J.A., Parrado-Hernández E. (2018). Partial Discharge Spectral Characterization in HF, VHF and UHF Bands Using Particle Swarm Optimization. Sensors.

